# Modelling of the Citric Acid Production from Crude Glycerol by Wild-Type *Yarrowia lipolytica* DSM 8218 Using Response Surface Methodology (RSM)

**DOI:** 10.3390/life12050621

**Published:** 2022-04-21

**Authors:** Romina Giacomobono, Roberto Albergo, Vito Valerio, Antonio Caporusso, Isabella De Bari

**Affiliations:** 1 Laboratory for Processes and Technologies for Biorefineries and Green Chemistry, Italian National Agency for New Technologies, Energy and Sustainable Economic Development (ENEA), C.R. Trisaia S.S. 106 Jonica, 75026 Rotondella, Italy; rominagiac@gmail.com (R.G.); vito.valerio@enea.it (V.V.); antonio.caporusso10@gmail.com (A.C.); isabella.debari@enea.it (I.D.B.); 2 School of Agricultural, Forest, Food and Environmental Sciences, University of Basilicata, Viale dell’Ateneo Lucano 10, 85100 Potenza, Italy

**Keywords:** bioconversion, oleaginous yeast, biorefinery, citric acid, crude glycerol, Response Surface Methodology, *Yarrowia lipolytica*

## Abstract

Crude glycerol is the main by-product of the biodiesel manufacturing industry (10% *w/w*). Its use as a substrate in microbial fermentations is a concrete strategy to efficiently address its market surplus. In this study, the conversion of crude glycerol to citric acid, a key biochemical in the emerging bioeconomy, by a wild-type yeast *Yarrowia lipolytica* DSM 8218 was modelled using the Response Surface Methodology. The model relates C/N mass ratio and crude glycerol concentration to maximize the citric acid yield in flask scale using two different N sources, yeast extract and ammonium sulphate. Under the optimal conditions (yeast extract, C/N 141, glycerol 33 g/L), the conversion yield was 0.249 g/g. The optimal conditions were used for up-scaling a fed-batch fermentation in a 2 L bioreactor highlighting a metabolic shift from mannitol to citric acid when high stirring rates were applied (800 rpm). In these conditions, a morphic transition from pseudo-mycelial form to round-shaped yeast-like cells was observed too.

## 1. Introduction

The global environmental concerns we are facing nowadays along with the world population being expected to reach 9.7 billion people by 2050 (United Nations 2019) highlight the urgent need to drastically change the current economy model, based on an inappropriate use of natural resources and crude materials. The transition to a new, competitive, and sustainable circular economy model must include the closing loop of any production process and the abolition of waste generation. Indeed, a great research effort is still made to develop any useful technology and solution to reduce and reuse all the secondary products and wastes coming from industrial processes. For this purpose, microbial biotechnology can significantly contribute to the development of biorefineries as many wild type and engineered microorganisms are able to produce relevant value-added biomolecules and biochemicals from waste materials.

Among all, crude glycerol is one of the most abundant industrial wastes with promising applications in the biorefinery sector. It is the main side-product of the biodiesel industry where it is generated in 10% (*w/w*) during the transesterification reaction [1]. As a result of the rapid growth of the biodiesel production, during the last two decades, an increasing surplus of unrefined glycerol flooded the chemical market, making it necessary to develop new applications for this bulk material. Both its purification and disposal are still economically disadvantageous for the producers due to the low price of refined glycerine. This issue has therefore encouraged the biodiesel and oleochemical companies themselves to set up efficient technologies to transform crude glycerol into valuable products and sell them, to stay competitive [2]. Besides being a chemical platform for chemical transformations, crude glycerol can be used as a feedstock for microbial fermentations as several microorganisms including bacteria, yeasts and fungi have shown the ability to consume and convert it to value-added bioproducts. For example, anaerobic bacteria belonging to *Clostridium*, *Enterobacter* and *Lactobacillus* species [3] or *Klebsiella* *pneumoniae* [4] can be employed to convert glycerol to 1,3-propanediol, a valuable chemical used as a solvent in the cosmetic industry. Alternatively, glycerol has been used as a sole substrate to produce ethanol by *E. coli* strains [5], but attempts to produce organic acids [6], polyols [7] and lipids as Single Cell Oils (SCOs) [8] are also common in the literature. The present study was mainly focused on the bioconversion of crude glycerol to citric acid (CA) through yeast fermentation. CA is indeed an attractive biochemical, considered to be one of the most economically feasible products of the microbiological industry due to its wide applications as an acidulant, food preservative, drug stabilizer and chelating agent. Traditionally, CA is produced through *A. niger* fermentation providing sugar molasses as substrate [9], but the process requires multiple stages and expensive raw materials. Thus, alternative production processes like bacteria and/or yeast fermentation using inexpensive substrates are under investigation.

Especially, over time yeasts have been even preferred to fungi and bacteria due to their higher resistance to substrate concentration and to metal ions, easy cultivation and higher fermentation rates. In addition, yeasts are easier to genetically modify [10], so strains can be engineered to increase CA production. Looking at the literature, *Yarrowia lipolytica* is a well-known workhorse for CA production; for example, the *Y. lipolytica* 1.31 strain on crude glycerol (200 g/L) produced about 124.5 g/L of CA, accounting for 0.62 g/g of CA yield [11]. Furthermore, a 0.78 g/g yield of CA was obtained with the mutant *Y. lipolytica* *Wratislavia* AWG7 on raw glycerol. [12] On the other hand, the mutant strain *Yarrowia lipolytica* NG40/UV5 was tested on several renewable carbon substrates (rapeseed oil, glucose, glycerol, ethanol, glycerol-containing waste of biodiesel industry and glucose-containing aspen waste). The best condition (1.5 g/g CA yield) was obtained on rapeseed oil [13].

The aim of this present study is to test a wild-type strain, namely, *Y. lipolytica* DSM 8218 since few papers in the literature investigated its metabolic properties and conversion ability [14] and no papers have been published so far concerning its application to produce CA. Moreover, even if belonging to Yarrowia genera, this specific strain has never been tested for citric/isocitric acid production, nor its ability to grow on raw glycerol has ever been proven. In the literature, it has been widely demonstrated that many strains of *Y. lipolytica* produce both citric and isocitric acid (ICA) [15,16], but as this study is still in a preliminary stage, only the CA production has been considered in the mathematical model.

The crude glycerol concentration and C/N mass ratio have been tested: crude glycerol concentration as it might impair yeast growth and CA production due to its impurities content (methanol, residual salts and soaps) like other fermentation procedures [6] and C/N ratio because of the deficiency of nitrogen source compared to the carbon one [9] might induce the accumulation and secretion of CA because of the metabolic imbalance.

In order to evaluate the individual and collective effect of C/N mass ratio and crude glycerol concentration on CA production, the Response Surface Methodology (RSM) with a Central Composite Design (CCD) has been applied through a 3-level Full Factorial Design of Experiment that allows to gather major information on the combined effect of the tested parameters compared to the traditional “one-factor at a time” strategy.

Finally, the optimized conditions obtained from the RSM mathematical model were applied to a fed-batch fermentation test in a 2 L bioreactor in order to verify the scalability of the process and to preliminary assess any effect of airflow and stirring on the metabolites production.

## 2. Materials and Methods

### 2.1. Crude Glycerol

The crude glycerol (GLY) used in this study was kindly provided by an Italian biodiesel plant, Ital Bi Oil s.r.l. The same batch of crude glycerol has been used for all the experiments, whose composition is (in %) glycerol 90.5, acetate 6.1, moisture 2.7, ash 2.6 and methanol 0.95.

### 2.2. Yeast Strain and Growth Conditions

The wild-type strain *Yarrowia lipolytica* DSM 8218 (DSMZ, Braunschweig, Germany) was used in this study. This strain has been isolated from a diesel tank and it is naturally able to grow on fuels making it interesting to investigate its ability to grow on raw glycerol containing potential microbial inhibitors and residues of the diesel manufacturing. It was grown on YPD agar (Sigma Aldrich, St Louis, MO, USA) plates for 48 h at 30 °C prior inoculation to the fermentation medium. The basal fermentation medium had the following composition in elements (g/L): KH_2_PO_4_ 5, K_2_HPO_4_ 1, MgSO_4_·7H_2_O 1, CaCl_2_·2H_2_O 0.15, FeSO_4_·7H_2_O 0.004, ZnCl_2_·7H_2_O 0.04, MnSO_4_·H_2_O 0.076 and CuSO_4_·5H_2_O 0.001. As a carbon source, either pure glycerol (99%, Sigma Aldrich) or crude glycerol was used with a final concentration in the medium of 30 g/L for preliminary tests. GLY concentrations of 15, 27.5 and 40 g/L were tested during the RSM optimization procedure. Likewise, Yeast Extract (YE) or Ammonium Sulphate (AS) were alternatively provided as nitrogen source, to test the following C/N mass ratio (g/g): 200, 110, 20, considering that nitrogen content in YE is 10%. All media were autoclaved at 121 °C for 15 min prior inoculation. In media containing AS, a filter sterilized vitamin solution was added, with the following final composition (g/L): thiamine 0.5, riboflavin 0.04, niacin 0.5, pantothenic acid 0.685, pyridoxine 0.05, biotin 0.0009, folic acid 0.002 and cobalamin 0.001. The pH was kept >6.0. Every fermentation test was conducted in triplicate in 500 mL Erlenmeyer flask with 50 mL of working volume and inoculated with 5 mL of a stationary phase pre-culture grown on YPD (50 g/L) for 72 h and then kept at 30 °C on a rotary shaker at 200 rpm, for 165 h.

### 2.3. Optimization of the Fermentation Medium by RSM

Through the software Design-Experiment^®^, version 10.01 (Stat-Ease Inc., Minneapolis, MN, USA), RSM was used to easily optimize the fermentation medium composition to maximize CA yield (YCA) measured as g of CA produced/g of glycerol supplied. A 3-level Full Factorial design was applied to relate the effect of two independent variables, namely crude glycerol concentration (A, *X*_1_) and C/N ratio (B, *X*_2_), on YCA. For statistical calculations, the variables *X_i_* were coded as xi according to Equation (1)

*x_i_* = *X_i_* − (*X_oi_*/Δ*X_i_*) (1)

where *x_i_* is the coded value of the *i^th^* factor, *X_i_* the natural value, *X_oi_* the value at the center point, Δ*X_i_* the step change value.

As displayed in Table 1, three levels of each variable were tested: GLY concentration (15, 27.5, 40 g/L) and C/N ratio (200, 110, 20).

Two distinct Design of Experiment with the same setup (Table 2) were performed to alternatively test two nitrogen sources (YE and AS).

The central point was replicated three times resulting in a total of 11 runs for each experimental design. YCA was selected as the dependent output variable. Biomass yield (Y_BM_) was also calculated as g of dry biomass/g of glycerol supplied and considered a dependent variable for speculations. The second-degree polynomials (Equation (2)) were calculated with the statistical package (Stat-Ease Inc., Minneapolis, MN, USA) to estimate the response of the dependent variable Y (Y_CA_ and Y_BM_)

*Y* = *b*_0_ + *b*_1_*x*_1_ + *b*_2_*x*_2_ + *b*_11_*x*_1_^2^ + *b*_22_*x*_2_^2^ + *b*_12_*x*_1_*x*_2_ (2)

where *Y* is the predicted response; *x*_1_ and *x*_2_ are the independent variables; *b*_0_ is the offset term; *b*_1_ and b_2_ are the linear effects; *b*_11_ and *b*_22_ are the squared effects, and *b*_12_ is the interaction term. The statistical analysis was done by ANOVA (one-way analysis of variance).

### 2.4. Scale Up to 2 L Bioreactor

A fed-batch fermentation was performed in a 2-L Biostat B^®^ (Sartorius AG, Gottingen, Germany) bioreactor, equipped with two Rushton turbines. The working volume was 1.5 L. The fermentation conditions were 30 °C, pH 5.5 ± 0.01 (NaOH 5 M, H_2_SO_4_ 2 M), glycerol 33 g/L and C/N 141 using YE as nitrogen source. After the sterilization (121 °C, 20 min), the vessel was inoculated with 150 mL of a stationary phase pre-culture grown on YPD (50 g/L) for 72 h. Incoming airflow was filtered through a 0.2 μm filter (Sartorius, Midisart 2000). The bioreactor was allowed to run for 256 h, and the substrate was fed when glycerol was almost exhausted to restore the initial conditions. Airflow and agitation rate were set as follows before and after the feed: airflow 3 L/min and agitation 200 rpm in the first phase (158 h), airflow 1 L/min and agitation rate 800 rpm in the second phase.

### 2.5. Analytical Techniques

Yeast growth was followed by measuring the optical density at λ = 600 nm (OD 600) and by measuring the biomass dry weight: 1 mL of the culture broth was centrifuged at 4000 rpm for 10 min, and the yeast cells were washed twice with water and dried in an oven at 60 °C to a constant weight.

To analyze the total amount of glycerol and the main metabolites of interest in the medium, CA, ICA, acetic acid (AA) and mannitol (Mann), the culture broth was centrifuged at 4000 rpm for 10 min, and 1 mL of the supernatant was properly diluted with water. The concentration of the metabolites was measured on an HPIC-chromatograph Dionex ICS5000 ((Dionex, Thermo Scientific, Waltham, MA, USA) on a Nucleogel ION 300 OA mixed-mode column. The operating conditions were the following: the refractive index detector (mod. Shodex RI101) was set at 40 °C, while the column was operated with 2.5 mM H_2_SO_4_ as mobile phase, at 60 °C and 0.400 mL/min flow rate.

Diagnostic kit (Roche Diagnostics GmbH, Mannheim, Germany) was used for the assay of ICA concentrations. The quantification of the ICA was based on the measurement of the NADPH produced from isocitrate dehydrogenase, during the conversion of the ICA to α-ketoglutarate.

## 3. Results and Discussion

**Figure 1:**
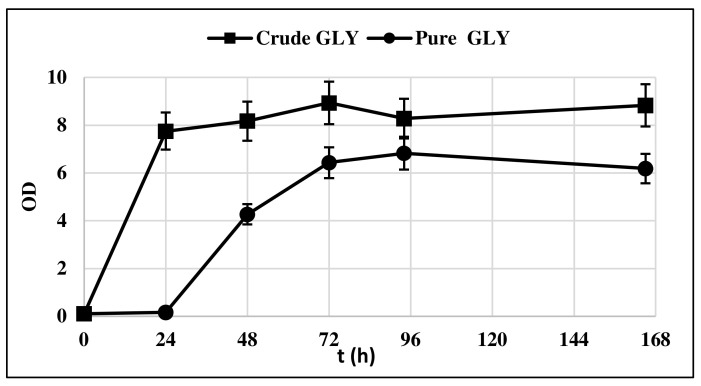
*Y. lipolytica* DSM 8218 growth profile on pure and crude glycerol. The presented data represent the mean and the standard deviation (≤10%) of four biological replicates.

The strain *Y. lipolytica* DSM 8218 was firstly assessed for its ability to grow on crude glycerol. The yeast was cultivated in 500 mL flasks under nitrogen deficiency, that is, with a C/N equal to 100. Pure and crude glycerol 30 g/L were solely used as carbon sources with the aim of assessing any impairing effect of crude glycerol impurities content on yeast growth. Figure 1 compares the biomass (BM) growth curve of *Y. lipolytica* on crude and pure glycerol.

**Figure 2:**
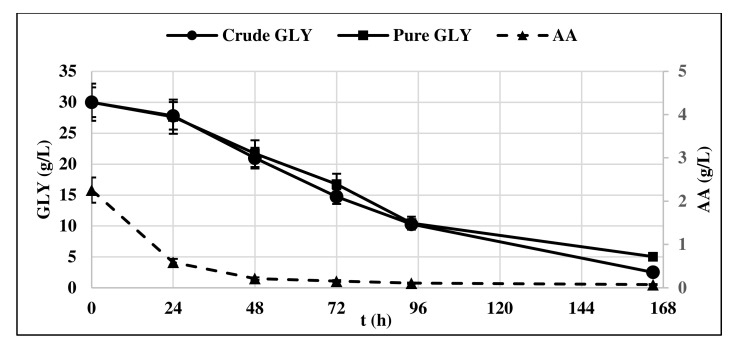
Pure and crude glycerol (GLY) consumption profile of *Y. lipolytica* DSM 8218. The presented data represent the mean and the standard deviation (≤10%) of four biological replicates.

The lag phase was longer when pure glycerol was used as substrate, whereas crude glycerol resulted in a faster yeast growth, as at t = 24 h, it had already entered the exponential growth phase. This could be probably due to the presence of AA in crude glycerol, which is consumed first in the culture medium (Figure 2).

Indeed, oleaginous yeasts including *Y. lipolytica* can use different carbon sources including acetic acid and convert them to lipids [17,18,19].

This is due to its naturally high flux capacity for acetyl-CoA that is required for lipid efficient lipid accumulation [20]. Moreover, the concentration of methanol in this study has been calculated to be 0.009 mol/L, considering the GLY concentration applied (30 g/L) and the content of methanol in the provided crude glycerol (0.95%). This is about 10 times lower than the minimum concentration of methanol required to inhibit cell growth of a wild-type strain of *Y. lipolytica* as estimated by Vartiainen et al. [21].

Furthermore, Samul et al. [22] reported some cases in which the impurities content in crude glycerol did not affect the CA production, with final conversion yields very close to or better than pure glycerol.

**Figure 3:**
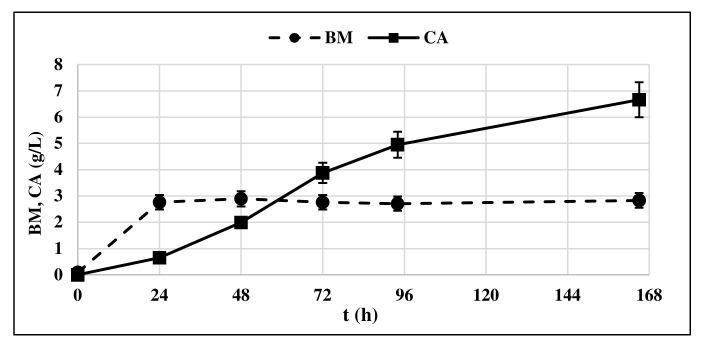
Citric acid (CA) production and biomass accumulation (BM) profile of *Y. lipolytica* DSM 8218 grown on crude glycerol. The presented data represent the mean and the standard deviation (≤10%) of four biological replicates.

The production of CA by *Y. lipolytica* cultured on GLY was observed starting from t = 24 h (Figure 3) in correspondence with the start of the stationary phase of yeast growth, confirming what was already shown in the literature [13,23].

CA concentration in the medium increased over time, along with the consumption of GLY and ended up in a final concentration of 6.33 g/L. Very low concentrations of ICA were detected, minor than 5% respect to the produced CA produced. For this reason, the ICA content was no longer determined in subsequent experiments. Biomass production remained constant at 3.3 g/L all along the production phase.

Once assessed the ability of the selected strain to grow on crude glycerol without any impairment and to produce CA, the study further proceeded for the optimization of the fermentation parameters through the Response Surface Methodology (RSM) [24,25] using Central Composite Design [26].

The variables and the values of levels were selected from a literature-based review: a large range of C/N ratio (20–200) was explored to find the optimal value to achieve the highest CA yield; by contrast, a relatively narrow range of GLY concentration (15–40 g/L) was investigated to keep the contaminants content of GLY from affecting the cell viability. The variables which most influence the production and secretion of CA to the extracellular medium were identified and selected, namely, nitrogen deficiency in presence of excess of the carbon source [27] and the concentration of the carbon substrate itself [28]. All crude glycerol samples were extracted from a single batch as described in the materials and methods section. In fact, since Dobrowolski et al. [23] demonstrated that different batches of crude glycerol originate different growth patterns, using the same batch for all tests, any distortion in the model due to the use of different types of crude glycerol has been avoided.

Two parallel designs were performed alternatively using Yeast Extract (YE) and Ammonium Sulphate (AS) as nitrogen source. AS has been included in the experimental design as it is significantly cheaper than YE, so that its use at the industrial scale would cut the process costs.

The experiments were carried out as per the designs, namely, 11 for each design. The CA obtained after 165 h fermentation under different combinations of variables was estimated (Table 3 and Table 4), as well as biomass accumulation and yield.

During the fermentation experiments, the consistent production of Mann as an additional metabolite was revealed, and thus, its concentration in the medium is reported too. By applying multiple regression analysis on the experimental data, the following second order polynomial equations were found to represent the CA production adequately, when using AS (Equation (3)) YE (Equation (4)), respectively:

Y_CA_ = −0.20597 + 0.010142 *X*_1_ + 1.24188 *E* − 003 *X*_2_ + 5.83555 *E* − 006 *X*_1_*X*_2_ − 1.38764 *E* − 004 *X*_1_^2^ − 4.0157 *E* − 006 *X*_2_^2^ (3)

Y_CA_ = −0.19891 + 0.013609 *X*_1_ + 3.39728 *E* − 003 *X*_2_ + 2.15327 *E* − 005 *X*_1_*X*_2_ −2.61815 *E* − 004 *X*_1_^2^ − 1.43541 *E* − 005 *X*_2_^2^ (4)

where *X*_1_ stands for crude glycerol concentration, and *X*_2_ stands for C/N ratio.

At the preliminary statistical evaluation of the mathematical model of Y_CA_ response (Equation (3)) obtained with AS performed with ANOVA, it was found that: F-value = 19.03 (significant, *p*-value = 0.0029) and Lack-of-Fit = 32.06 (significant, *p*-value = 0.0299). Nonetheless, to consider the model statistically valid, the Lack-of-Fit coefficient must be not significant. Using the diagnostic tool, it emerged that run 1 and 7 were outliers in the analysis of Cook’s distance, a commonly used estimate of the influence of a data point when performing a least-squares regression analysis [29]. The software provides that outliers can be ignored in the model, starting from the farthest one: thereby ignoring run 7, the ANOVA results (Table 5) changed resulting in Lack-of-Fit = 9.17 (not significant, *p*-value = 0.0983).

Therefore, the model could be considered statistically valid, even though the interaction term AB resulted to be not significant, with a *p*-value of 0.27.

The same procedure was applied to the mathematical model (Equation (4)) generated with YE. In this case, initially the ANOVA statistical analysis stated that F-value was 69.60 (*p*-value = 0.0001) and Lack-of-Fit = 31.56 (*p*-value = 0.0309). The diagnostic procedure was applied and run 11 turned out to be an outlier within the comparison residuals vs. predicted at the analysis of Externally Studentized Residuals (the quotient resulting from the division of a residual by an estimate of its SD excluding the improbably large cases). By ignoring run 11 in the DoE design setup, the ANOVA coefficients turned into: F-value = 500.81 (significant, *p*-value < 0.0001) and Lack-of-Fit = 3.06 (not significant, *p*-value = 0.2465) as indicated in Table 6.

A Lack-of-Fit close to 1 means that there is a linear relationship between the two variables, that is, there is no lack of fit in the regression model. Therefore, the model was a good fit, and the interaction term itself AB was significant (*p*-value 0.0009). YE was consequently selected as the nitrogen source to further proceed with the optimization procedure, considering also the doubled CA yields obtained compared to AS (Table 3 and Table 4).

The reduced yield obtained with the use of the inorganic nitrogen source could be attributed to a suboptimal vitamin formulation used in the medium, since *Y. lipolytica* is known for being auxotrophic for thiamine, an essential cofactor for pyruvate dehydrogenase and other TCA cycle enzymatic activity. Cavallo et al. [30] reported that *Y. lipolytica* is not able indeed to synthesize the thiamine pyrimidine backbone, which is a limiting factor to produce CA.

**Figure 4:**
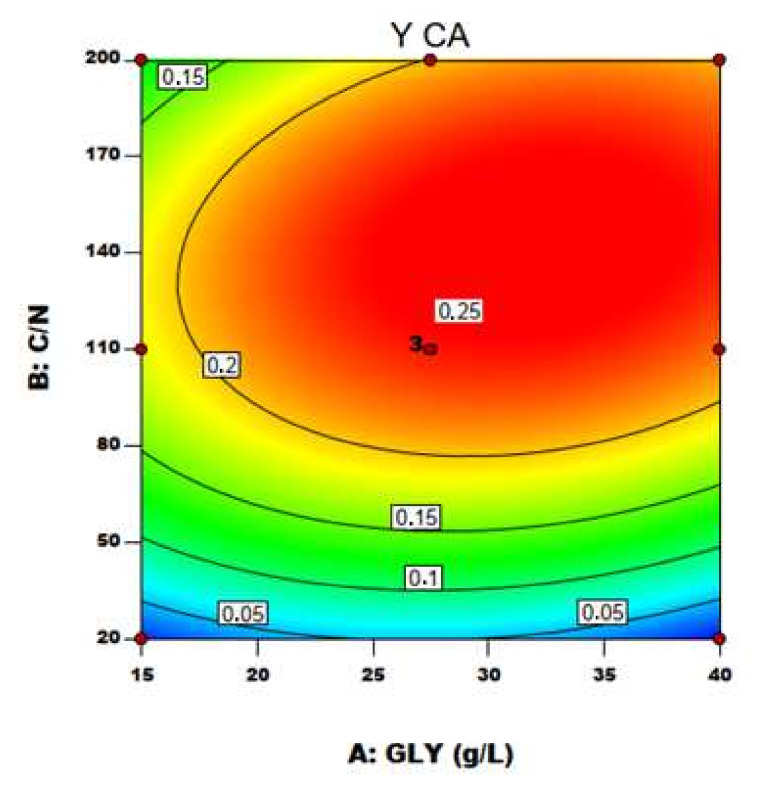
Contour plot of C/N ratio and crude glycerol concentration (GLY) effects on CA yield using YE as nitrogen source.

**Figure 5:**
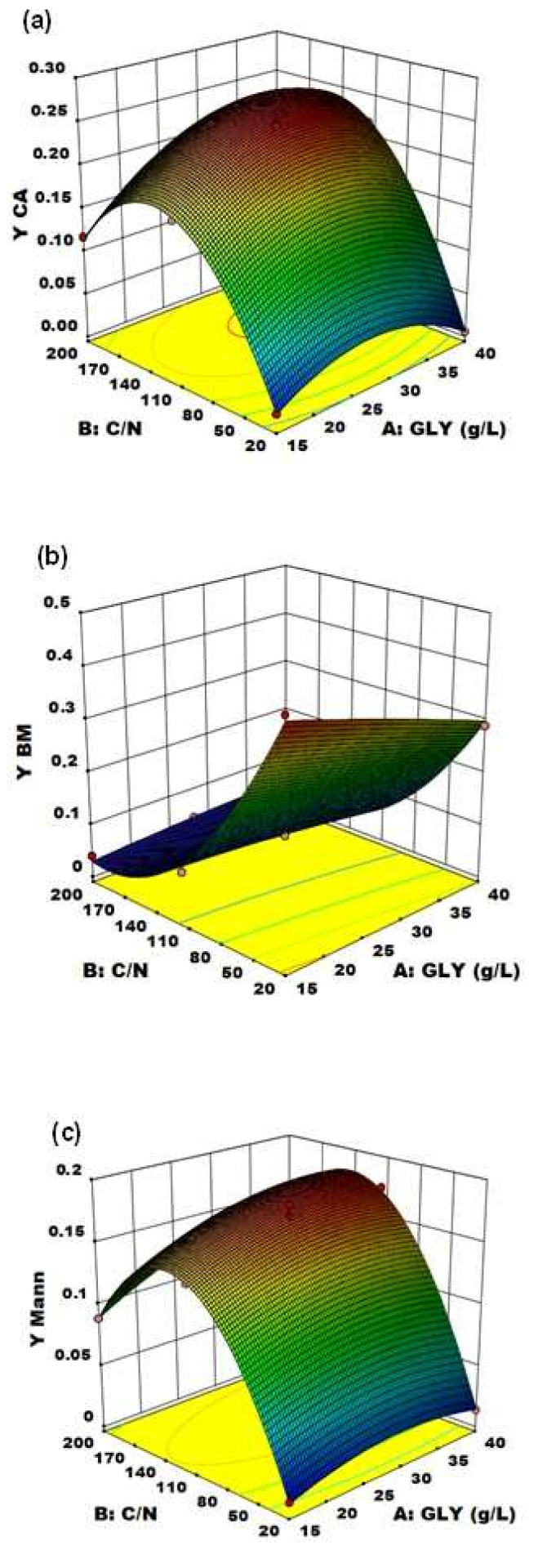
Response surface of (**a**) C/N ratio and crude glycerol concentration (GLY) effects on CA yield using YE as nitrogen source, (**b**) crude glycerol concentration (GLY) and C/N ratio effects on biomass production yield using YE as nitrogen source and (**c**) crude glycerol concentration (GLY) and C/N ratio effects on mannitol production yield using YE as nitrogen source.

With the established model by applying the regression analysis of Equation (4), the optimum level of C/N ratio and GLY concentration were predicted along with the associated optimum CA yield. Figure 4 and Figure 5 represent, respectively, the contour plot and the response surfaces for the optimization of fermentation parameters to produce CA, BM and Mann using YE as nitrogen source in the medium.

The effect of the two variables on CA production is clearly shown in Figure 5a: particularly, C/N was confirmed to be the major driving force in *Y. lipolytica* DSM 8218 in determining CA accumulation, as found in the literature for other *Y. lipolytica* strains [9,15]. An increase in crude glycerol concentration itself at a fixed low C/N ratio (20) did not result in any significant effect on CA production. An increase in C/N ratio along with crude glycerol concentration pushed instead CA yield to a maximum level found within the experimental range. On an equal crude glycerol concentration (40 g/L), an increase in C/N from 110 to 200 resulted in a 24% increase of CA yield.

Alongside, an opposite effect on BM production and yield was evidenced (Figure 5b). Particularly, BM and CA accumulation turned out to be two inversely related processes: this is demonstrated by the specularity between the two response surfaces. At low C/N [30], biomass accumulation was favored (reaching up to Y_BM_ = 0.436 g of biomass per g of glycerol consumed) at the expense of CA production which was virtually absent; whereas, at high C/N (>110), CA accumulation in the culture medium started immediately after the cessation of cell growth, when nitrogen was supposed to be totally consumed.

**Figure 6:**
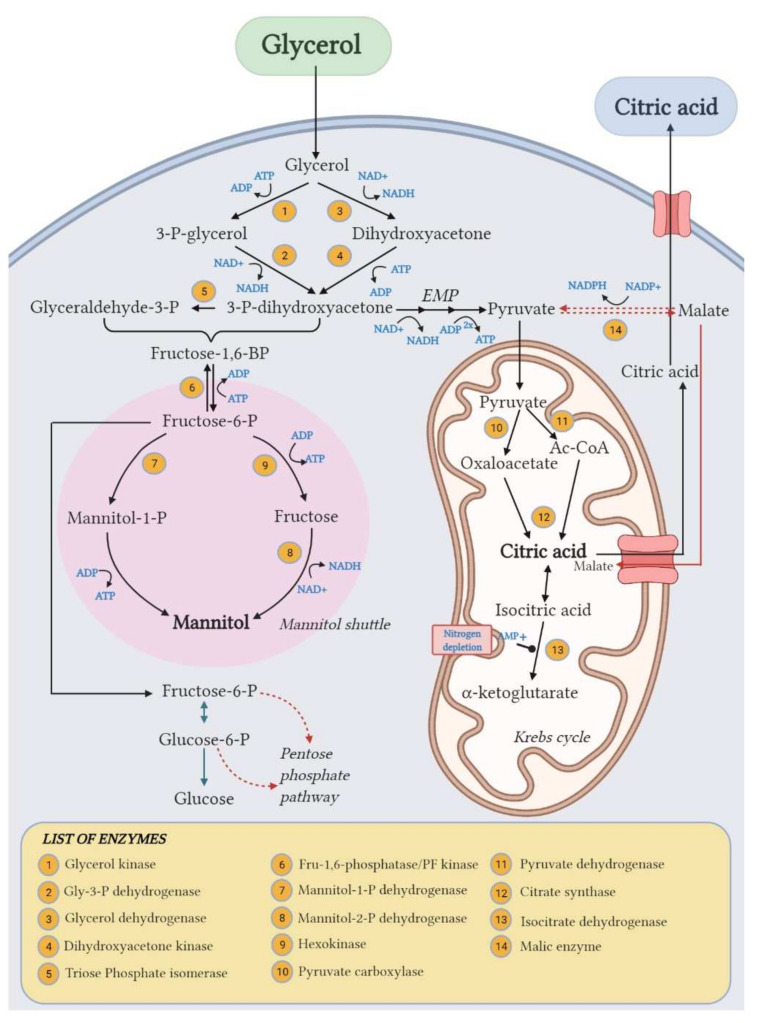
Proposed pathways for CA and mannitol biosynthesis from glycerol in *Y. lipolytica*. Created with BioRender.com.

On the other side, the unexpected significant accumulation of Mann (up to 17.4% yield) along with CA highlighted the wide metabolic flexibility of *Y. lipolytica* in response to different fermentation conditions. The response surface of Mann yield (Figure 5c) shows the same trend of the CA yield one, with a Mann yield increase along with increasing C/N ratio. Moreover, in accordance with Rzechonek et al. [15], the production of Mann increases when the pH lowers due to the production of organic acids during the fermentation. Crude glycerol concentration showed a minor effect in this case. Although the biosynthetic pathways leading to the production of these two metabolites (Figure 6) are not related [27], the production of Mann in *Y. lipolytica* had already been reported in the literature [15,31].

This yeast species is indeed known to be an osmophilic yeast, able to produce polyols (mainly Mann and/or erythritol) under osmotic pressure, and thus, the presence of salts in the crude glycerol used in the fermentation medium in addition to the osmotic power of glycerol itself can explain the production of Mann in this case. In some studies, the addition of glycerol [32] or surfactants [7] is used as an inducer of polyols biosynthesis in osmophilic yeasts like *Y. lipolytica*.

Notwithstanding the simultaneous production of additional metabolites, the equation model was used to predict the optimum level of the tested variables to get the higher CA yields. The optimum fermentation conditions for higher CA production can be attained at: GLY concentration 32.57 g/L and C/N ratio 141. When these optimal conditions were applied in the confirmation test, the maximum CA yield of 0.249 g/g was obtained. This value was not significantly different from the predicted 0.259 g/g (α = 0.05). In addition, 0.18 g of Mann per g of GLY consumed was produced. All the values are reported in Table 7.

Therefore, the mathematical model generated by the DoE software turned out statistically valid and reliable in describing the interaction among the tested factors and the response.

This value corresponds to the 35% of the maximum theoretical yield of CA from glycerol, which is estimated to be 0.70 g/g, calculated assuming all glycerol provided is converted to CA. Yeast metabolism implies that glycerol entry to central carbon metabolism can occur via two pathways (conversion to DHA followed by phosphorylation or phosphorylation to G-3-P followed by oxidation) both resulting in the production of DHAP. DHAP is then converted to PEP and pyruvate subsequently. The pyruvate dehydrogenase catalyzes the formation of a molecule of acetyl-coA which reacts with oxaloacetate in the TCA cycle, forming a molecule of CA (citrate synthase). Considering that 1 mole of CA derives from 3 moles of acetyl-coA, a total of 3 moles of glycerol (92.1 g/mol × 3 = 276.3 g) are needed to generate 1 mole of CA (192.1 g). Following this approach, the maximum theoretical yield would be 0.70 g/g. However, in the literature, yields of 0.9 g/g were achieved with some mutant strains [9]: in this case, intra-cellular gluconeogenesis and various anaplerotic reactions that generate CA cycle precursors or intermediates can potentially increase the product yield. Furthermore, carbon losses due to the tropophase and substrate consumption for the cell maintenance must be taken into account as they contribute to lower the maximum achievable yield of CA. Krzystek et al. [33] calculated the theoretical CA yield in *A. niger* by sucrose including the maintenance coefficients due basal cellular activities which resulted to be 0.80 mol per mol of sucrose and experimentally found that CA production reached the 83% of the maximum theoretical yield. In addition, it could be hypothesized that not all the accumulated CA was secreted to the extracellular medium: the active transport system is indeed influenced by various parameters such as air saturation, pH, temperature, medium composition and growth state of cells [34]. A simultaneous quantification of the intracellular CA would confirm the role of the secretion step to optimize the production yield.

In the present paper, the maximum Y_CA_ obtained (0.249 g/g) was lower than those reported by other authors when crude glycerol was used as substrate in flask fermentation experiments with wild type *Y. lipolytica* strains: 0.46–0.59 g/g in Sarris et al. [35], 0.52 g/g in Papanikolaou et al. [36].

Under optimized conditions favoring CA accumulation, 18% of the substrate was converted to Mann, which is another potentially attractive bioproduct. On the whole, a total of 46% of the substrate consumed was transformed into two main value-added useful products (CA and Mann). This metabolic pattern is similar to other *Y. lipolytica* strains [36]; however, the ratio between the two metabolites slightly differs from other wild-type strains isolated in natural environments. Egermeier et al. [37] showed that strains isolated from cheese and butter and the lab-strain H222 mainly converted crude glycerol to polyols, whereas *Y. lipolytica* W29, isolated from sewage, mostly produced CA.

The optimized conditions (glycerol 33 g/L, C/N 141 and YE as Nitrogen source) were then further applied to a fed-batch fermentation test in a 2 L bioreactor to verify the scalability of the process and to preliminary assess any effect of oxygenation on the metabolites production.

The concentration of dissolved oxygen (DO) is an important parameter to take in account for the CA production [38] and generally for the production of organic acids such as isocitric acid [15,39], pyruvic acid [40] and α-Ketoglutaric Acid [41]. Ferreira et al. [42] showed that raising the oxygen volumetric mass transfer coefficient (Kla), the conversion of crude glycerol by *Y. lipolytica* W29 to CA increased of 700%. For *Y. lipolytica* strains Wratislavia 1.31 and AWG7, the maximum CA yields were reported at DO of 40% of saturation [43]. Moreover, the DO influence on the CA final concentration has been studied in relation to different carbon sources used. Controlling the DO at 50% of saturation significantly increases CA production on glucose and glucose/glycerol media, while it has no effect on a pure glycerol-based medium [44].

**Figure 7:**
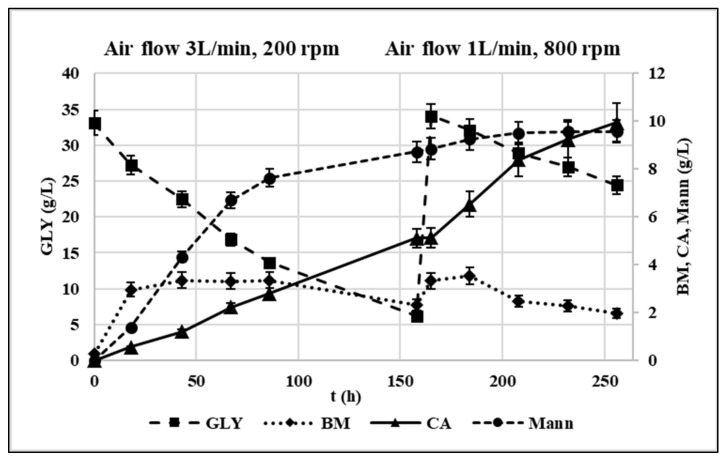
Fed-batch experiment profile in terms of GLY consumption and CA, mannitol and biomass production in g/L. Initial conditions: crude glycerol 33 g/L, C/N 141. Oxygenation parameters: airflow 3 L/min, 200 rpm (t = 0–158 h); airflow 1 L/min, 800 rpm (t = 158–256 h). pH = 5.5, T = 30 °C.

The process data are shown in Figure 7.

Up to 158 h, airflow and agitation rate were set at 3 L/min and 200 rpm, respectively. 5.1 g/L CA were produced corresponding to a production of CA 38% lower compared to the corresponding flask trial. The metabolic yield (i.e., grams of produced CA/grams of consumed GLY) was 0.19 g/g while the production rate 0.048 g/L/h. At the same time, the production of Mann was higher than in shaken flasks corresponding to a metabolic yield of 0.32 g/g. When fresh substrate was fed to restore the initial conditions, the oxygenation parameters were changed as follows: the airflow was lowered to 1 L/min, and the agitation rate was increased to 800 rpm according to Rzechonek et al. [15]. This enabled to avoid an excessive turbulence in the vessel. Under these conditions, the CA production rate sped up to 0.084 g/L/h, and its metabolic yield increased in this phase up to 0.53 g/g while the *Y. lipolytica* culture remains almost stable for the entire duration of the fermentation in accordance with Rymowicz et al. [16]. This evidence suggested a positive effect of high agitation rates on the overall oxygenation and on CA production by *Y. lipolytica*. Similarly, Rywinska et al. [43] found that by increasing the agitation rate from 400 to 800 rpm, the CA yield passed from 0.09 g/g to 0.47 g/g. Moreover, the reduction of Mann production by 86% was observed corresponding to a metabolic yield of 0.18 g/g.

Although few studies focused on the regulation of Mann production from GLY containing media by *Y. lipolytica* [12], its biosynthetic reaction is known to be involved in NAD^+^ regeneration: under oxygen limitation conditions, NADH in excess cannot be oxidized through oxidative phosphorylation, and thus, the synthesis of Mann from fructose-6-P allows to regain NAD^+^ [27]. Therefore, the increase in Mann production observed in the first phase can likely be attributed to the oxygen limitation due to low agitation rate, which also reduced the CA productivity. Oppositely, the increase in agitation rate from 200 to 800 rpm improved the overall media oxygenation, favoring CA accumulation at the expense of Mann. This metabolic pattern is comparable to what observed for *Y. lipolytica* W29 in Papanikolaou et al. [45] for which a metabolic shift towards CA production was observed in bioreactor trials, while insignificant polyol quantities were produced.

In Table 8 and Table 9, a comparison with literature studies of conversion yields to CA by several *Y. lipolytica* strains when crude glycerol and other carbon sources are used has been provided for bioreactor experiments.

Considering that the specific yeast strain here tested (*Y. lipolytica* DSM 8218) has never been specifically investigated by other authors for this process, it can be stated that 0.53 g/g is a preliminary but promising result in terms of CA metabolic yield.

**Figure 8:**
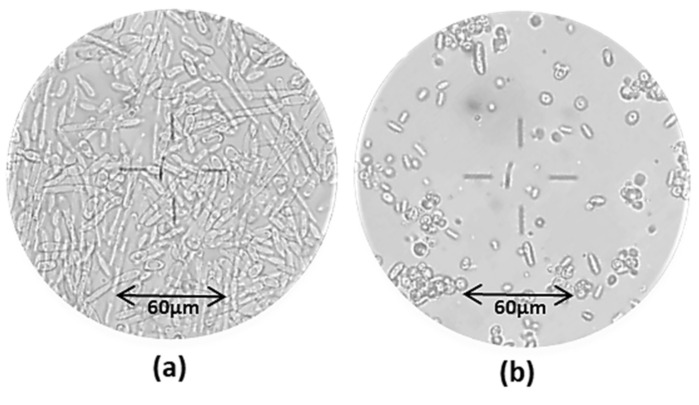
Microscopic observation (20×) of the cell culture broth at different times: prevalence of mycelial cell forms at t = 67 h (**a**) under low agitation rate (200 rpm); prevalence of yeast-like cells at t = 208 h (**b**) under high agitation rate (800 rpm).

Figure 8 shows the microscopic observations of the culture medium in the two oxygenation set-ups.

The images highlighted the predominance of long mycelial cells at t = 67 h, i.e., in the first phase, whereas round-shaped yeast-like cells were predominant at t = 208 h, under a higher agitation rate. *Y. lipolytica* species are known to be natural dimorphic fungi which can be found in the yeast-like, mycelial or pseudo-mycelial form according to the environmental conditions including pH, temperature, oxygenation, nutritional status and presence of fatty materials [56]. Several authors [43,57] reported the predominance of the mycelial and/or pseudo-mycelial form under limited oxygen conditions. This finding confirms that the first set-up determined a low oxygen uptake, and this was responsible for a low CA production.

Overall, this experiment proved the influence of oxygenation set-up on CA production and highlighted its essential role to optimize the process efficiency.

## 4. Conclusions

The Response Surface Methodology (RSM) was successfully applied to optimize the citric acid (CA) production from crude glycerol by *Y. lipolytica* DSM 8218.

This strain grew faster on crude glycerol than on pure glycerol, due to acetic acid consumption. The mathematical model was reliable: the optimized parameters were crude glycerol 33 g/L and C/N 141, and the best nitrogen source was identified in the yeast extract. Under these conditions, *Y_CA_* was 0.249 g/g, corresponding to 35% of the maximum theoretical yield. Mannitol was produced too, but further studies are required to assess whether its production is advantageous or competitive. The scale-up process highlighted the essential role of mixing and the oxygenation control strategy. A high agitation rate (800 rpm) favors CA accumulation and promotes a morphic transition from the pseudo-mycelial form to round-shaped yeast-like cells.

## Figures and Tables

**Table 1:** Experimental design range and levels of the factors of crude glycerol concentration (GLY) and carbon to nitrogen ratio (C/N).

Code	Factors	Unit	Range and Levels		
			−1	0	+1
A	GLY	g/L	15	27.5	40
B	C/N		20	110	200

**Table 2:** General design setup and runs scheduled for each Design of Experiment (YE and AS).

Run	Coded Variable		Level	
	A	B	A	B
1	+1	+1	40	200
2	+1	−1	40	20
3	0	0	27.5	110
4	0	0	27.5	110
5	+1	0	40	110
6	−1	+1	15	200
7	−1	−1	15	20
8	0	+1	27.5	200
9	0	0	27.5	110
10	−1	0	15	110
11	0	−1	27.5	20

**Table 3:** Results of shake flask experiments performed with *Yarrowia lipolytica* on different glycerol concentrations, ranging from 14 to 40 g/L; Yeast Extract (YE) as nitrogen source at different C/N ratios, ranging from 20 to 200, in terms of biomass (BM) accumulation, citric acid (CA) and mannitol (Mann) production (g/L), and citric acid yield (Y_CA_).

RUN	GLY	C/N	BM g/L	CA g/L	Mann g/L	Y_CA_ g/g
	*X*_1_	*X*_2_				
1	40	200	1.92 ± 0.02	8.98 ± 0.01	6.02 ± 0.20	0.207
2	40	20	12.10 ± 1.30	0.23 ± 0.08	0.52 ± 0.09	0.006
3	27.5	110	2.17 ± 0.32	6.57 ± 0.32	4.82 ± 0.20	0.245
4	27.5	110	2.96 ± 0.32	7.18 ± 0.32	5.13 ± 0.20	0.240
5	40	110	3.46 ± 0.01	9.76 ± 0.05	7.78 ± 0.17	0.219
6	15	200	0.69 ± 0.03	2.02 ± 0.32	1.52 ± 0.02	0.119
7	15	20	6.73 ± 1.82	0.21 ± 0.01	0.13 ± 0.02	0.015
8	27.5	200	1.33 ± 0.08	5.94 ± 0.30	3.99 ± 0.30	0.195
9	27.5	110	2.47 ± 0.32	7.30 ± 0.32	4.64 ± 0.20	0.247
10	15	110	1.40 ± 0.06	2.80 ± 0.18	2.23 ± 0.16	0.178
11	27.5	20	8.23 ± 0.52	nd	0.25 ± 0.05	0

**Table 4:** Results of shake flask experiments performed with *Yarrowia lipolytica* on different glycerol concentrations, ranging from 14 to 40 g/L, Ammonium Sulphate (AS) as nitrogen source at different C/N ratios, ranging from 20 to 200, in terms of biomass (BM) accumulation, citric acid (CA) and mannitol (Mann) production (g/L), and citric acid yield (Y_CA_).

RUN	GLY	C/N	BM g/L	CA g/L	Mann g/L	Y_CA_ g/g
	*X*_1_	*X*_2_				
1	40	200	1.91 ± 0.09	4.52 ± 0.07	4.67 ± 0.08	0.109
2	40	20	7.45 ± 0.12	nd	0.06 ± 0.04	0
3	27.5	110	2.69 ± 0.11	2.10 ± 0.16	4.18 ± 0.18	0.074
4	27.5	110	2.68 ± 0.11	2.08 ± 0.15	4.30 ± 0.09	0.073
5	40	110	3.77 ± 0.22	4.20 ± 0.08	7.51 ± 0.13	0.100
6	15	200	0.74 ± 0.03	0.32 ± 0.01	0.97 ± 0.01	0.023
7	15	20	3.17 ± 0.15	nd	0.040 ± 0.001	0
8	27.5	200	1.58 ± 0.11	2.51 ± 0.16	3.69 ± 0.11	0.088
9	27.5	110	2.72 ± 0.11	1.87 ± 0.14	4.09 ± 0.12	0.068
10	15	110	1.52 ± 0.09	0.15 ± 0.04	1.55 ± 0.13	0.01
11	27.5	20	6.39 ± 0.24	nd	0.010 ± 0.001	0

**Table 5:** ANOVA results of statistical evaluation of Y_CA_ (obtained in DoE experiments with AS as nitrogen source), after ignoring run 7.

Source	Sum of Squares	Df	Mean Square	F Value	*p*-Value Prob > F	
Model	0.016	5	3.165 × 10^−3^	66.48	0.0006	significant
A-GLY	5.149 × 10^−3^	1	5.149 × 10^−3^	108.14	0.0005	
B-C/N	7.236 × 10^−3^	1	7.236 × 10^−3^	151.98	0.0002	
AB	7.791 × 10^−5^	1	7.791 × 10^−5^	1.64	0.2700	
A^2^	9.820 × 10^−4^	1	9.820 × 10^−4^	20.63	0.0105	
B^2^	2.210 × 10^−3^	1	2.210 × 10^−3^	46.42	0.0024	
Residual	1.904 × 10^−4^	4	4.761 × 10^−5^			
Lack of Fit	1.717 × 10^−4^	2	8.586 × 10^−5^	9.17	0.0983	not significant
Pure Error	1.872 × 10^−5^	2	9.361 × 10^−6^			
Cor Total	0.016	9				

**Table 6:** ANOVA results of statistical evaluation of Y_CA_ (obtained in DoE experiments with YE as nitrogen source) response model, after ignoring run 11.

Source	Sum of Squares	Df	Mean Square	F Value	*p*-Value Prob > F	
Model	0.074	5	0.015	500.81	<0.0001	significant
A-GLY	2.334 × 10^−3^	1	2.334 × 10^−3^	78.55	0.0009	
B-C/N	0.025	1	0.025	844.22	<0.0001	
AB	2.347 × 10^−3^	1	2.347 × 10^−3^	78.98	0.0009	
A^2^	3.124 × 10^−3^	1	3.124 × 10^−3^	105.11	0.0005	
B^2^	0.027	1	0.027	893.73	<0.0001	
Residual	1.189 × 10^−4^	4	2.972 × 10^−5^			
Lack of Fit	8.958 × 10^−5^	2	4.479 × 10^−5^	3.06	0.2465	not significant
Pure Error	2.930 × 10^−5^	2	1.465 × 10^−5^			
Cor Total	0.075	9				

**Table 7:** Results and statistical analysis of the confirmation test performed at optimized conditions: GLY = 32.57 g/L C/N = 141. The prediction interval (PI) was set at 95%.

Response Statistical Data							
Resp.	Predicted Mean	Observed Mean	Std Dev	n	SE Pred	PI Low	PI High
Y_CA_	0.259 g/g	0.249 g/g	0.005	3	4.18 × 10^−3^	0.25	0.27
Y_Mann_	0.180 g/g	0.179 g/g	0.003	3	3.99 × 10^−3^	0.18	0.19

**Table 8:** Comparative data of yield conversion of crude glycerol to CA when *Y. lipolytica* strains are used in bioreactor experiments.

*Y. lipolytica* Strain	Wild Type	Y_CA_ (g/g)	Reference
DSM 8218	yes	0.53	This study
NG40/UV7	no	0.64	[9]
ACA-DC 50109	yes	0.45–0.62	[36]
Wratislavia AWG7	no	0.63	[43]
Wratislavia AWG7	no	0.78	[12]
Wratislavia AWG7	no	0.52	[46]
Wratislavia AWG7	no	0.46	[11]
1.31	no	0.62	[11]
K-1	no	0.40	[11]
AJD pADUTGut1/2	no	0.51	[15]
A-101-1.22	no	0.60–0.77	[16]
NCYC 3825	no	0.17	[47]

**Table 9:** Comparative data of yield conversion of various carbon sources to CA when *Y. lipolytica* strains are used in bioreactor experiments.

*Y. lipolytica* Strain	Wild Type	Substrate	Y_CA_ (g/g)	Reference
H222	yes	glucose	0.55	[48]
VKM Y2373	yes	glucose	0.75	[49]
W29	yes	wet corn syrups	0.24	[30]
DSM 3286	yes	plant oils	0–0.36	[50]
ACA-YC 5033	yes	olive mill wastewaters	0.61	[51]
XYL+	no	xylose	0.53	[52]
H222-S4 (p67ICL1)	no	sucrose	0.75–0.82	[53]
AWG7 INU8	no	inuline	0.85	[54]
NRRL-Y-1095	yes	n-paraffins	0.4–0.99	[55]
NG40/UV5	no	ethanol	0.85	[13]

## Data Availability

Not applicable.
